# Tumor-Associated Fibronectin Targeted Liposomal Nanoplatform for Cyclophilin A siRNA Delivery and Targeted Malignant Glioblastoma Therapy

**DOI:** 10.3389/fphar.2018.01194

**Published:** 2018-10-17

**Authors:** Phei Er Saw, Ao Zhang, Yan Nie, Lei Zhang, Yingjie Xu, Xiaoding Xu

**Affiliations:** ^1^Guangdong Provincial Key Laboratory of Malignant Tumor Epigenetics and Gene Regulation, Medical Research Center, Sun Yat-sen Memorial Hospital, Sun Yat-sen University, Guangzhou, China; ^2^RNA Biomedical Institute, Sun Yat-sen Memorial Hospital, Sun Yat-sen University, Guangzhou, China; ^3^Department of Hepatobiliary Surgery, Sun Yat-sen Memorial Hospital, Sun Yat-sen University, Guangzhou, China; ^4^Department of Biochemistry and Molecular Cell Biology, Shanghai Key Laboratory for Tumor Microenvironment and Inflammation, Shanghai Jiao Tong University School of Medicine, Shanghai, China

**Keywords:** nanoparticle, aptide, siRNA, targeted delivery, glioblastoma

## Abstract

Malignant glioblastoma (GBM) is the most aggressive brain cancer that has a very low survival rate. With the rapid development of nanotechnology in the past few decades, the use of nanoparticles (NPs) for nucleic acid delivery is expected to have a revolutionary impact on GBM therapy. However, clinical success in GBM therapy remains a formidable challenge, mainly due to suboptimal *in vivo* delivery of therapeutics to glioma cells. Herein, we developed an aptamer-like peptide (aptide)-decorated liposomal nanoplatform for systemic small interfering RNA (siRNA) delivery and targeted GBM therapy. This nanoplatform is mainly composed of the following key components: (i) classic liposome structure with an aqueous core that can encapsulate therapeutic siRNA; (ii) hydrophilic polyethylene glycol (PEG) chains on the outer shell to prolong blood circulation; and (iii) surface-encoded aptide to specifically target the extra-domain B (EDB) of fibronectin that over-expressed on glioma cells. After systemic administration of these new siRNA delivery NPs, they can target the glioma cells and efficiently inhibit the GBM tumor growth by silencing the expression of cyclophilin A (CypA), which is up-regulated in brain cancer and plays an important role in malignant transformation of brain cancer and maintaining glioma cell stemness. These results suggest that the reported RNA interference (RNAi) NP platform herein could become an effective tool for targeted GBM therapy.

## Introduction

Glioblastoma (GBM) is the most common and aggressive form of brain cancer with poor diagnosis, difficult management, and low survival rate. Even when detected early, less than 5% of GBM patients are alive 5 years after diagnosis and the median survival rate is around 14.6 months (Behin et al., 2003; Brandsma and van den Bent, 2007; Johnson and O’Neill, 2012). Currently, multimodality treatment approach including surgical resection, radiotherapy, and chemotherapy is the standard care for GBM patients (Meyers et al., 2013; Cheng et al., 2014; Mahmoudi and Hadjipanayis, 2014; Krizbai et al., 2016). However, the development of resistance to therapies (e.g., radiotherapy and chemotherapy) has emerged as a persistent clinical problem and ultimately induces the failure of GBM treatment. Multiple reasons have been demonstrated to contribute to this disappointed outcome, including (i) the complex structure of the brain, (ii) the heterogeneous and invasive nature of GBM, and (iii) difficulty in delivering therapeutics specifically to glioma cells (Lesniak and Brem, 2004; Pardridge, 2005; Ozdemir-Kaynak et al., 2018; Shergalis et al., 2018). Therefore, there is a critical need to develop alternative strategies for more effective GBM treatment.

Since the discovery of RNA interference (RNAi) by Fire et al. (1998), RNAi technology has demonstrated significant potential for disease treatment by silencing the expression of target gene(s), especially those encoding “undruggable” proteins (Whitehead et al., 2009; Xu et al., 2016). However, the safe and effective delivery of RNAi agents such as siRNA to target cells remains a major hurdle for the widespread clinical application of RNAi technology. RNAi agents are biomacromolecules with polyanionic characteristics, which are easily attacked by serum nucleases and cannot readily cross cell membrane. Therefore, specific delivery vehicles are required to facilitate the intracellular uptake and cytosolic delivery of RNAi agents (Yang et al., 2012; Kanasty et al., 2013; Liu et al., 2017; Xu et al., 2017a,b). Over the past decades, nanoparticles (NPs) have been demonstrated as a powerful tool toward this end, especially showing hepatocyte-specificity in non-human primates and clinical trials (Zimmermann et al., 2006; Tseng et al., 2009; Jhaveri and Torchilin, 2014; Shi et al., 2017; Durymanov and Reineke, 2018). Nevertheless, it is still challenged to accomplish the systemic delivery of RNAi agents to a particular non-liver diseased tissue (e.g., solid tumor) and cell type, followed by sufficient intra-cytosolic transport. While several RNAi NP platforms have entered into early phase clinical trials for cancer treatment (Zuckerman and Davis, 2015), substantial obstacles still remain, including long blood circulation, selective accumulation at tumor site, and efficient tumor cell internalization. Specifically, these challenges are amplified by the structural complex of the brain (Pardridge, 2005; Mahmoudi and Hadjipanayis, 2014; Shergalis et al., 2018).

**FIGURE 1:**
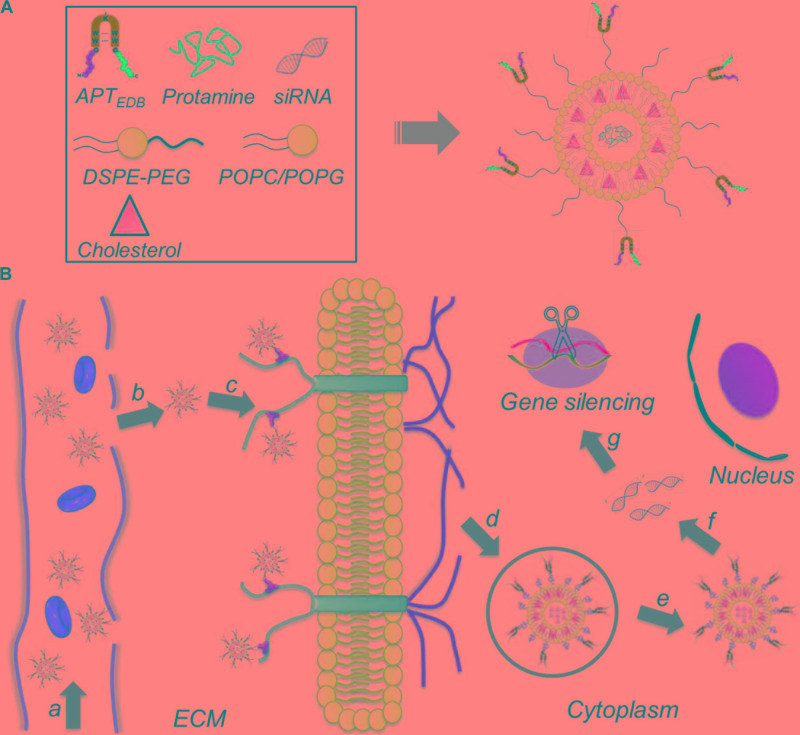
**(A)** Formulation of the aptide-decorated liposomal nanoplatform (APT-_EDB_ NPs); **(B)** schematic illustration of the APT-_EDB_ NPs for systemic siRNA delivery and targeted GBM treatment. After intravenous injection (a), the long-circulating NPs can accumulate in the GBM tumor tissues (b) and subsequently target the glioma cells via the specific recognition between aptide and EDB (c). After targeted cellular uptake (d,e), the APT-_EDB_ NPs can release the siRNA in the cytoplasm (f), leading to effective silencing of GBM-associated CypA expression and ultimate inhibition of GBM tumor growth (g).

To address these issues, we herein developed a liposome-based extra-domain B (EDB)-targeting nanoplatform for systemic siRNA delivery and GBM therapy. As shown in **Figure 1**, this nanoplatform is composed of a classic liposome structure (i.e., one phospholipid bilayer surrounding an aqueous core), hydrophilic polyethylene glycol (PEG) chains on the outer shell, and surface-encoded aptamer-like peptide (aptide) to specifically target the EDB of fibronectin that over-expressed on glioma cells (Borsi et al., 1992; Castellani et al., 2002; Han and Lu, 2017; Saw et al., 2017). After loading siRNA and then systemic administration, the resulting nanoplatform shows the following unique functions: (i) the hydrophilic PEG chains allow the NPs to escape immunological recognition, thus improving blood circulation; (ii) the surface-encoded aptide moieties can enhance the GBM targeting ability and intracellular siRNA delivery; and (iii) commercial available of the NP compositions and robust NP formulation enables the scale-up of this NP platform. As a proof of concept, we chose cyclophilin A (CypA) as a therapeutic target and systemically evaluated the EDB-targeting NPs for CypA siRNA (siCypA) delivery and its anticancer efficacy. CypA is a ubiquitously distributed protein belonging to the immunophilin family, which shows an activity of peptidylprolyl *cis trans* isomerase and plays an important role in regulation of protein folding (Wang and Heitman, 2005), trafficking (Shieh et al., 1989; Luban, 1996), assembly (Pan et al., 2008; Tanaka et al., 2011), immune-modulator and cell signaling (Jin et al., 2000; Satoh et al., 2008). It has been demonstrated that CypA is up-regulated in many cancers (e.g., liver, brain, and lung cancers) and is a key determinant for malignant transformation, epithelial to mesenchymal transition (EMT) and cancer metastasis (Yang et al., 2007; Qi et al., 2008). Recent research demonstrated that over-expressed CypA in GBM involves in maintaining glioma cell stemness via Wnt/β-catenin signaling pathway (Wang et al., 2017). Our *in vivo* results show that the systemic delivery of siCypA with the EDB-targeting NP platform can efficiently inhibit CypA expression in the tumor tissue and significantly inhibit GBM tumor growth.

## Materials and Methods

### Materials

CypA siRNA (siCypA) and Cy5.5-labeled CypA siRNA (Cy5.5-siCypA) were acquired from Dharmacon (United States). The siRNA sequences are as follows: 5′-UGA CUU CAC ACG CCA UAA UdTdT-3′ (sense); 5′-AUU AUG GCG UGU GAA GUC AdTdT-3′ (antisense). Protamine sulfate and sepharose CL-4B column were purchased from Sigma Aldrich (United States). 1-Palmitoyl-2-oleoyl-*sn*-glycero-3-phosphocholine (POPC), 1-palmitoyl-2-oleoyl-*sn*-glycero-3-phospho-(10-rac-glycerol) (POPG), 1,2-distearoyl-*sn*-glycero-3-phosphoethanolamine (DSPE), and PEG (2000)-DSPE (ammonium salt) (PEG_2000_-DSPE) were purchased from Avanti Polar Lipids (United States) and used as received. *N*-Maleimide-PEG_2000_-DSPE (ammonium salt) (Mal-PEG_2000_-DSPE) and plant cholesterol (Chol) were purchased from A.V.T. (Shanghai) Pharmaceuticals (China). EDB-targeting aptide (APT_EDB_) with an additional cysteine in the β-hairpin constant loop region (sequence from *N* to *C* terminal, CSSPIQGSWTWENGK(C)WTWGIIRLEQ) was synthesized by Guangzhou IGE biotechnology Co., Ltd (China). Lipofectamine 2000 (Lipo2000) was provided by Thermofisher Scientific (United States). Real-time PCR assay kit was procured from Promega (United States). All antibodies were purchased from Abcam (United States) and used according to the manufacturer’s protocol. All other chemicals were of reagent grade and used directly.

### Methods

#### Synthesis of APT_EDB_ Conjugated PEG_2000_-DSPE (APT_EDB_-PEG_2000_-DSPE)

The APT_EDB_-PEG_2000_-DSPE was synthesized via the reaction between the thiol group of APT_EDB_ and maleimide terminal group of Mal- PEG_2000_-DSPE. In brief, APT_EDB_ and Mal- PEG_2000_-DSPE were dissolved in dimethyl sulfoxide (DMSO) and chloroform, respectively. Subsequently, these two solutions were mixed in a molar ratio (APT_EDB_: Mal-PEG_2000_-DSPE) of 1:2. Under nitrogen atmosphere, the mixture was stirred at room temperature for 12 h. Thereafter, the mixture was transferred to dialysis membrane (MWCO 3500) and dialyzed against deionized water for 3 days. After freeze-drying under vacuum, the APT_EDB_-PEG_2000_-DSPE was collected as a white powder.

#### Preparation and Characterizations of EDB-Targeting siRNA-Loaded NPs

The classic rehydration method was employed to prepare the EDB-targeting siRNA-loaded NPs (Saw et al., 2015). POPC, Chol, and POPG were dissolved in chloroform in a molar ratio of 4:3:3 and APT_EDB_-PEG_2000_-DSPE (2.5 wt% of the total lipid) was then added. The mixture was stirred at room temperature for 10 min to form a homogenous solution. Subsequently, the solvent was removed by using rotary evaporator and a thin lipid film was thus generated. Then, HEPES-buffered 5% glucose (HBG) containing siCypA/protamine complexes was added and the resulting mixture (2 mg/mL) was briefly sonicated to accelerate the formation of siRNA-loaded liposomes. Thereafter, extrusions were performed by using a 100 nm polycarbonate membrane to ensure the formation of uniform liposomes, which were then passed over sepharose CL-4B column to obtain the purified siRNA-loaded NPs. To prepare the siRNA-loaded NPs without EDB-targeting ability, the POPC, Chol, and POPG were dissolved in chloroform in a molar ratio of 4:3:3 and then the siRNA-loaded NPs were prepared and purified according to the method described above.

The siRNA-loaded NPs were characterized in terms of size, zeta potential, and morphology. The particle size and zeta potential were examined by dynamic light scattering (DLS, Malvern Instruments Corporation). The morphology of siRNA loaded NPs was visualized by transmission electron microscopy (TEM, Tecnai G^2^ Spirit BioTWIN). To determine the siRNA encapsulation efficiency (EE), Cy5.5-siCypA was used to prepare the siRNA-loaded NPs according to the method aforementioned and the obtained NPs were dispersed in 1 mL of PBS. Subsequently, a small volume (5 μL) of the NP solution was withdrawn and mixed with 20-fold DMSO. The standard was prepared by mixing 5 μL of naked Cy5.5-siCypA solution with 20-fold DMSO. The fluorescence intensity of Cy5.5-siCypA was measured using a Synergy HT multimode microplate reader (BioTek Instruments), and the siRNA EE% is calculated as EE% = (*FI_NPs_* / *FI_Standard_*) × 100.

#### *In vitro* siRNA Release

Cy5.5-siCypA-loaded NPs were dispersed in 1 mL of PBS (pH 7.4) and then transferred to a Float-a-lyzer G2 dialysis device (MWCO 100 kDa, Spectrum) that was immersed in PBS (pH 7.4) at 37°C. At a predetermined interval, 5 μL of the NP solution was withdrawn and mixed with 20-fold DMSO. The fluorescence intensity of Cy5.5-siCypA was determined by Synergy HT multi-mode microplate reader.

#### Cell Culture

Human glioma cells (U87MG and U251MG), skin melanoma cells (A375), prostate cancer cells (PC3), breast cancer cells (MCF-7), and Jurkat leukemic T cells were incubated in DMEM medium with 10% FBS at 37°C in a humidified atmosphere containing 5% CO_2_.

#### Evaluation of the Expression of EDB and CypA in Glioma Cells

Real-time qPCR was used to evaluate the mRNA level of EDB and CypA in the glioma cells. The intracellular RNA was isolated with RiboEx using an RNA isolation kit (Geneall, South Korea). cDNA was then synthesized using reverse transcription technique using 1 μg of total RNA from each sample. The primers used for detecting the EDB domain of fibronectin were 5′-AAC TCA CTG ACC TAA GCT TT-3′ (forward) and 5′-CGT TTG TTG TGT CAG TGT AG-3′ (reverse). The primers for detecting the CypA were 5′-TAT CTG CAC TGC CAA GAC TGA GTG-3′ (forward) and 5′-CTT CTT GCT GGT CTT GCC ATT CC-3′ (reverse).

#### Targeted Cellular Uptake

Glioma cells (20,000 cells) were seeded in disks and incubated in 2 mL of DMEM medium containing 10% FBS for 24 h. Subsequently, the Cy5.5-siCypA-loaded NPs were added at a siRNA concentration of 5 nM, and the cells were allowed to incubate at 37°C for 4 h. After removing the medium and subsequently washing with PBS buffer thrice, the nuclei were stained with Hoechst 33342 and the cells were then viewed under a FV1000 confocal laser scanning microscope (CLSM, Olympus).

#### *In vitro* Gene Silencing

Glioma cells were seeded in 6-well plates (50,000 cells per well) and incubated in 2 mL of DMEM medium containing 10% FBS for 24 h. Subsequently, the cells were incubated with the siCypA-loaded NPs for 24 h. After washing the cells with PBS buffer thrice, the cells were further incubated in fresh medium for another 48 h. Thereafter, the cells were digested by trypsin and the intracellular RNA was isolated for real-time qPCR to examine the CypA expression. As a positive control, Lipo2000/siCypA complexes were prepared according to manufacturer’s protocol and then incubated with the glioma cells for 4 h. After washing the cells thrice with PBS, the cells were further incubated for another 48 h and then collected for real-time qPCR analysis.

#### *In vitro* Cell Viability Assay

Glioma cells were seeded in 96-well plates (5,000 cells per well) and incubated in 0.1 mL of DMEM medium with 10% FBS for 24 h. Thereafter, the siCypA-loaded NPs were added at predetermined concentration and the cells were allowed to incubate for 48 h. After removing the medium and washing the cells with PBS thrice, the cell viability was measured using the AlamarBlue assay according to the manufacturer’s protocol.

#### Animals

Healthy female BALB/c normal mice and nude mice (4–5 weeks old) were purchased from Sun Yat-sen University experimental animal center. All *in vivo* studies were performed in accordance with a protocol approved by the Institutional Animal Care and Use Committee at Sun Yat-sen University.

#### Pharmacokinetics

Healthy female BALB/c normal mice were randomly divided into three groups (*n* = 3) and given an intravenous injection of either (i) naked Cy5.5-siCypA, (ii) Cy5.5-siCypA-loaded NPs, or (iii) EDB-targeting NPs at a 1 nmol siRNA dose per mouse. At predetermined time intervals, 20 μL of blood was withdrawn and the wound was pressed for several seconds to stop bleeding. The fluorescence intensity of Cy5.5-siCypA in the blood was determined by Synergy HT multi-mode microplate reader. The blood circulation half-life (*t*_1/2_) was calculated according to previous report (Winter et al., 2013; Xu et al., 2017a).

#### GBM Xenograft Tumor Model

The tumor model was constructed by subcutaneous injection with 200 μL of glioma cell suspension (1:1 mixture of medium and Matrigel) with a density of 2 × 10^6^ cells/mL into the back region of healthy female nude mice. When the volume of the tumor xenograft reached ∼100 mm^3^, the mice were used for the *in vivo* experiments.

#### Evaluation of EDB Expression in GBM Xenograft Tumor Model

The tumor tissues were excised from the GBM xenograft tumor-bearing mice and then sectioned using Leica CM 1950 Research Cyrostat (Leica Biosystems, IL, United States) at a 5 μm thickness. Subsequently, the tumor sections were washed in PBS and fixed with 4% (w/v) paraformaldehyde. After blocking with 2% bovine serum albumin (BSA) for 1 h, the tumor sections were incubated with anti-BC-1 antibody (EDB specific antibody) overnight at 4°C. Then, the tumor sections were washed in PBS and Alexa Fluor 594-conjugated goat anti-mouse IgG was added. After 1 h incubation at room temperature, the tumor sections were washed with PBS thrice and mounted with DAPI containing mounting medium. The tumor sections were finally viewed under CLSM to examine the EDB expression.

#### Inhibition of Tumor Growth

GBM xenograft tumor-bearing nude mice were randomly divided into three groups (*n* = 4) and intravenously injected with (i) PBS, (ii) siCypA-loaded NPs or (iii) EDB-targeting NPs at a 1 nmol siRNA dose per mouse once every two days. All the mice were administrated four consecutive injections and the tumor growth was monitored every two days by measuring perpendicular diameters using a caliper and tumor volume was calculated as follows:

$$V=W^{2}\times L/2$$

where W and L are the shortest and longest diameters, respectively.

#### Histology

After the aforementioned treatment, the mice in each group were sacrificed at end of the evaluation period, and the tumor tissues and main organs (heart, liver, spleen, lung, and kidney) were collected. After fixing with 4% paraformaldehyde and then embedding in paraffin, the tissue was sectioned and stained with hematoxylin-eosin (H&E) and then viewed under an optical microscope. In addition, the apoptosis in the tumor tissues was examined by terminal deoxynucleotidyl transferase dUTP nick-end labeling (TUNEL) assay according to manufacturer’s protocol.

## Results and Discussion

### Preparation and Characterizations of the NPs

EDB of fibronectin has been demonstrated to be highly expressed in the aggressive and malignant GBM (Borsi et al., 1992; Castellani et al., 2002). Therefore, EDB-targeting nanoplatform could be used a robust vehicle to deliver various therapeutics for GBM treatment. Arising from the excellent targeting ability of aptamer, we previously developed aptamer-like peptide (aptide) and demonstrated its strong ability to specifically bind EDB with high affinity (*K*_d_ ∼16 nM) (Saw et al., 2013, 2015, 2017). Based on the high EDB expression in GBM and strong targeting ability of aptide, we herein conjugated the EDB-targeting aptide (APT-_EDB_) to PEG_2000_-DSPE, which was then formulated with other lipids (POPC, Chol, and POPG) to obtain the APT-_EDB_-decorated liposomes (denoted APT-_EDB_ NPs) for the systemic siRNA delivery and targeted GBM treatment (**Figure 1**). In this work, we employed this nanoplatform to deliver siCypA because it can specifically silence the expression of cancer-associated CypA that over-expressed in GBM (Yang et al., 2007; Qi et al., 2008).

**FIGURE 2:**
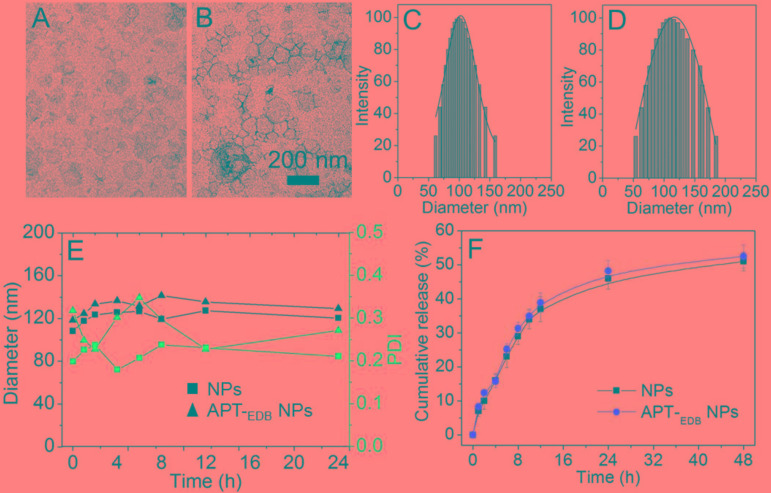
**(A–D)** TEM images **(A,B)** and size distribution **(C,D)** of the siCypA-loaded NPs without aptide decoration **(A,C)** and the APT-_EDB_ NPs **(B,D)**. **(E)** Size and polydispersity (PDI) of the siCypA-loaded NPs with (APT-_EDB_ NPs) or without aptide decoration (NPs) incubated in PBS buffer containing 10% FBS for different time. **(F)** Cumulative siRNA release from the Cy5.5-siCypA-loaded NPs with (APT-_EDB_ NPs) or without aptide decoration (NPs) incubated in PBS buffer at 37°C.

**Figure 2** shows the morphology of the siCypA-loaded APT-_EDB_ NPs. Similar as other reported liposomes (Saw et al., 2013, 2015, 2017), the APT-_EDB_ NPs show a spherical morphology with an average size of ∼112 nm determined by DLS (**Figure 2D**). Compared to the NPs without APT-_EDB_ decoration (∼104 nm, **Figure 2C**), there is around 10 nm increase after APT-_EDB_ decoration, suggesting the success in the APT_EDB_ decoration because APT-_EDB_ shows a hydrodynamic size of ∼5 nm (Kim et al., 2012). Moreover, due to the presence of PEG chains on the outer layer, these siRNA-loaded NPs show a high stability. As shown in **Figure 2E**, either with or without APT-_EDB_ decoration, possibly due to the presence of weak interaction between the particles and protein, there is a slight change in the particle size and polydispersity density (PDI) when incubating the NPs in 10% FBS-containing PBS solution for 8 h. However, this interaction will become stable as the incubation time increases and therefore there is no obvious size change after 8 h incubation. After obtaining these stable NPs, we next labeled the siCypA with fluorescent dye of Cy5.5 to examine the siRNA encapsulation efficiency (EE%) and release behavior. Through analyzing the fluorescence intensity, the EE was determined as ∼90%. The siRNA release profile is shown in **Figure 2F**. As can be seen, the NPs show a sustained siRNA release behavior. Around 35% of loaded siRNA can be released within 12 h and the cumulative release reaches ∼50% 48 h later.

### Determination of EDB and CypA Expression

**FIGURE 3:**
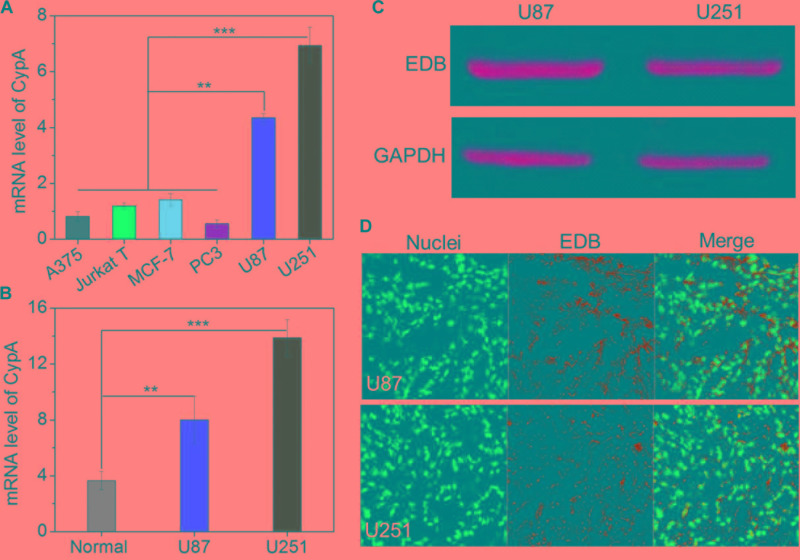
**(A)** The mRNA level of CypA in the skin melanoma cells (A375), Jurkat leukemic T cells, prostate cancer cells (PC3), breast cancer cells (MCF-7), and glioma cells (U87 and U251). **(B)** The mRNA level of CypA in the normal brain tissues and GBM xenograft tumor tissues of nude mice. **(C)** Western blot analysis of the EDB expression in the glioma cells (U87 and U251). **(D)** Immunofluorescence analysis of the EDB expression in the GBM xenograft tumor tissues of nude mice. The nuclei and EDB were stained with blue and green fluorescence, respectively. ^∗∗^*P* < 0.01; ^∗∗∗^*P* < 0.001.

The main purpose of this work is to develop EDB-targeting siRNA delivery nanoplatform for GBM treatment by silencing the CypA expression. Prior to evaluating the gene silencing efficacy of the APT-_EDB_ NPs, we first examined the CypA and EDB expression in the glioma cells. We chose different cancer cell lines and examined the corresponding CypA expression using real-time qPCR. As shown in **Figure 3A**, compared to skin melanoma cells (A375), Jurkat leukemic T cells, prostate cancer cells (PC3), and breast cancer cells (MCF-7), glioma cells (U87 and U251) show a much higher CypA expression. This encouraging result suggests that CypA is organ-specific over-expressed. To further verify this result, we compared the CypA expression in U87 and U251 xenograft tumors with normal brain tissues. As can be seen, the U87 or U251 xenograft tumors show around twofold or fourfold higher CypA expression that that of normal brain tissues (**Figure 3B**), implying that silencing CypA expression can be used as a potential strategy for GBM treatment.

After validation of the high CypA expression in the glioma cells, we next examined the EDB expression on these cells. As shown in **Figure 3C**, western blot analysis demonstrates that both U87 and U251 cells have a high EDB expression. Moreover, if using these two cell lines to construct xenograft tumor model, EDB is also highly expressed in the tumor tissues (green fluorescence, **Figure 3D**). All these results are consistent with previous reports (Yang et al., 2007; Qi et al., 2008) and indicate that EDB is indeed a suitable biomarker for targeted GBM therapy. Combining with results of PCR analysis (**Figures 3A,B**), because U251 cells show a relatively higher level of CypA and EDB expression compared to U87 cells, we chose U251 cells to evaluate the targeting and gene silencing ability of the APT-_EDB_ NPs.

### Evaluation of GBM-Targeting Ability and *in vitro* Gene Silencing

**FIGURE 4:**
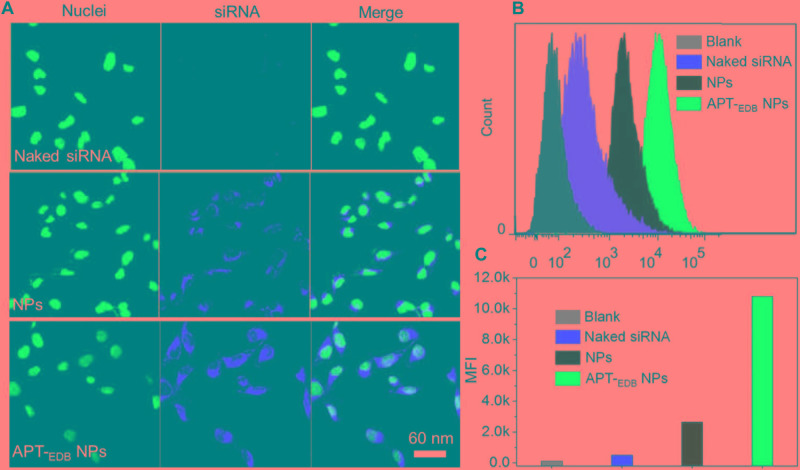
**(A)** Fluorescent images of U251 cells incubated with the Cy5.5-siCypA-loaded NPs with (APT-_EDB_ NPs) or without aptide decoration (NPs) for 4 h. The nuclei and siRNA were labeled with blue and red fluorescence, respectively. **(B,C)** Flow cytometry analysis **(B)** and mean fluorescence intensity [MFI, **(C)**] of U251 cells incubated with the Cy5.5-siCypA-loaded NPs with (APT-_EDB_ NPs) or without aptide decoration (NPs) for 4 h.

The GBM-targeting ability was evaluated by incubating U251 cells with the siCypA-loaded NPs. From the fluorescent images shown in **Figure 4A**, compared to naked siRNA, higher amount of siRNA can be observed and distributed in the cytoplasm of the cells incubated with the siRNA-loaded NPs. More importantly, due to the presence of specific recognition between aptide and EDB, the cellular uptake of APT-_EDB_ NPs is much higher than that of the NPs without aptide decoration. This result is further proven by the flow cytometry analysis. As shown in **Figure 4B**, U251 cells show stronger ability to internalize the APT-_EDB_ NPs and intracellular mean fluorescence intensity (MFI) is more than fivefold stronger than the cells incubated with the NPs without aptide decoration (**Figure 4C**).

**FIGURE 5:**
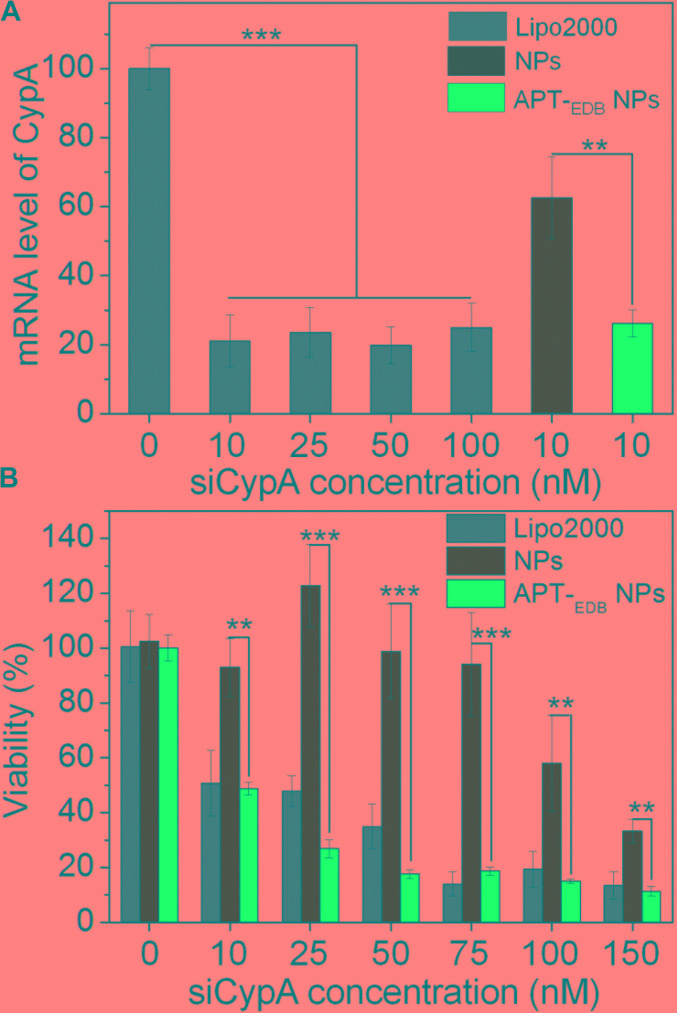
**(A)** The mRNA level of CypA in U251 cells treated with the Lipo2000/siCypA complexes at various siRNA concentrations, and the siCypA-loaded NPs with (APT-_EDB_ NPs) or without aptide decoration (NPs) at a 10 nM siRNA concentration. **(B)** Viability of U251 cells treated with the Lipo2000/siCypA complexes, and the siCypA-loaded NPs with (APT-_EDB_ NPs) or without aptide decoration (NPs) at different siRNA concentrations. ^∗∗^*P* < 0.01; ^∗∗∗^*P* < 0.001.

After validation of the GBM-targeting ability of the APT-_EDB_ NPs, we next examined their *in vitro* gene silencing efficacy. **Figure 5A** shows the mRNA level of CypA in U251 cells treated with the siCypA-loaded NPs. With the GBM-targeting ability to improve the cellular uptake, the APT-_EDB_ NPs shows an effective gene silencing and there is around 80% decrease in the mRNA level of CypA in the cells at a siRNA concentration of 10 nM. This high gene silencing efficacy is similar as the commercial available Lipo2000 and is more than twofold higher than that of the NPs without aptide decoration. With this suppressed CypA expression, the cells have a very low viability. As shown in **Figure 5B**, lower than 50% of the cells are alive when treated with the APT-_EDB_ NPs at a siRNA concentration of 10 nM. In contrast, at the same siRNA concentration, around 90% of the cells are still alive when treated with the NPs without aptide decoration. This result highlights the importance of the GBM-targeting ability of the siRNA-loaded NPs to their gene silencing in glioma cells.

### Evaluation of *in vivo* Anti-tumor Efficacy

**FIGURE 6:**
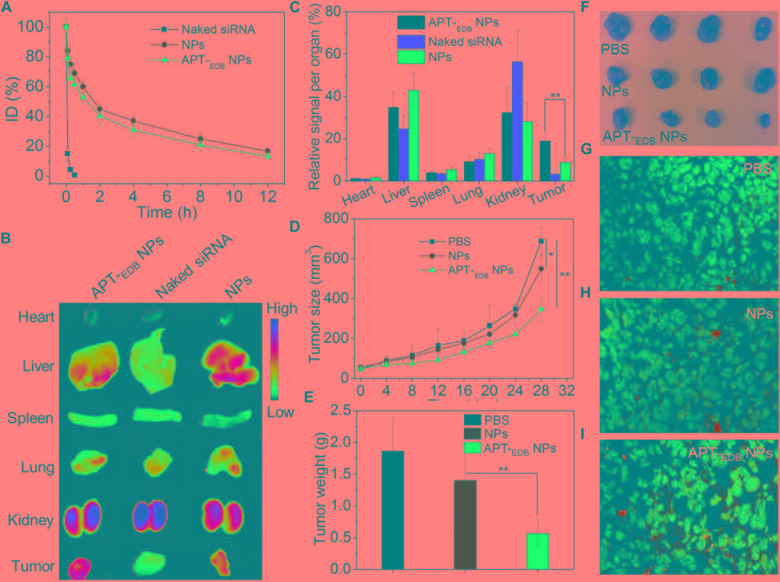
**(A)** Blood circulation profile of the naked Cy5.5-siCypA and the Cy5.5-siCypA-loaded NPs with (APT-_EDB_ NPs) or without aptide decoration (NPs). **(B)** Overlaid fluorescent image of the main organs of the GBM xenograft tumor-bearing mice treated with naked siRNA, and the siCypA-loaded NPs with (APT-_EDB_ NPs) or without aptide decoration (NPs). **(C)** Biodistribution of the siRNA obatined from **(B)**. **(D)** Tumor growth profile of the GBM xenograft tumor-bearing mice treated with PBS, and the siCypA-loaded NPs with (APT-_EDB_ NPs) or without aptide decoration (NPs). Intravenous injections are indicated by the arrows. **(E,F)** Weight **(E)** and representative photograph **(F)** of the tumor tissues from the mice in each group after 28 day evaluation period. **(G–I)** TUNEL staining of the collected tumor tissues in **(F)**. TUNEL-positive apoptotic cells were stained with green fluorescence. ^∗^*P* < 0.05; ^∗∗^*P* < 0.01.

Encouraged by the strong EDB-targeting ability and effective gene silencing of the APT-_EDB_ NPs, we finally evaluated their *in vivo* anti-tumor efficacy. The pharmacokinetics was first examined by intravenously injecting the Cy5.5-siCypA-loaded NPs to normal adult mice. As shown in **Figure 6A**, the naked siRNA is rapidly cleared from the blood and its half-life (*t_1/2_*) is less than 10 min. In contrast, the APT-_EDB_ NPs show much longer blood circulation with blood *t_1/2_* of around 4.68 h, which is comparable to the *t_1/2_* of the NPs without aptide decoration (∼5.12 h). This long circulation feature is mainly attributed to protection by the PEG chains on the outer layer (Knop et al., 2010) and will ensure the accumulation of the APT-_EDB_ NPs in the tumor tissues via enhance and permeable retention (EPR) effect (Bertrand et al., 2014). The biodistribution results in **Figures 6B,C** also demonstrate our statement. As shown in **Figure 6B**, the siRNA loaded NPs show higher tumor accumulation than that of naked siRNA. Moreover, due to the presence of APT-_EDB_ targeting ligand, the APT-_EDB_ NPs show more than twofold higher tumor accumulation than that of the NPs without aptide decoration (**Figure 6C**).

**FIGURE 7:**
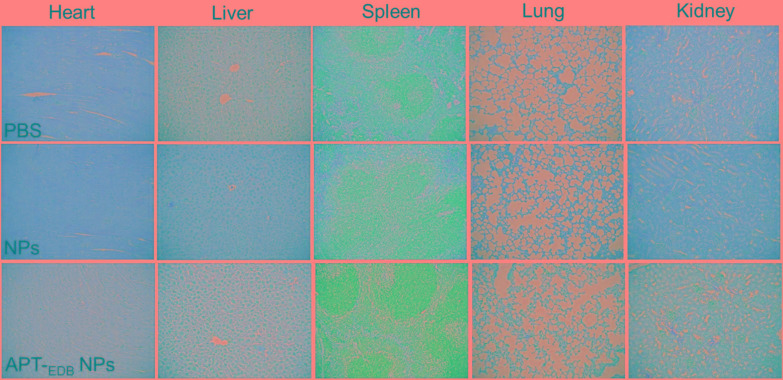
Histological section of the main organs of the GBM xenograft tumor-bearing mice after systemic treatment with PBS, and the siCypA-loaded NPs with (APT-_EDB_ NPs) or without aptide decoration (NPs). H&E; magnification 100×.

The *in vivo* anti-tumor efficacy was examined by intravenous injection of the siCypA-loaded APT-_EDB_ NPs to GBM xenograft tumor-bearing mice once every two days at a 1 nmol siRNA dose per mouse. As shown in **Figures 6D–F**, after four consecutive injections, the tumor growth is obviously inhibited compared to the mice treated with PBS or NPs without aptide decoration. There is around sevenfold increase in the tumor size (from ∼50 to ∼350 mm^3^) (**Figure 6B**). However, for the mice treated with PBS or the NPs without aptide decoration, there is around 14-fold (from ∼50 to ∼690 mm^3^) or 11-fold (from ∼50 to ∼550 mm^3^) increase in the tumor size. This tendency is further supported by the result of TUNEL assay. From the images shown in **Figures 6G–I**, more apoptotic cells can be observed in the tumor section of the mice treated with the siCypA-loaded APT-_EDB_ NPs (**Figure 6I**). Noting that, the administration of the NPs does not induce apparent *in vivo* toxicity. As shown in **Figure 7**, no noticeable histological changes can be found in the tissues from heart, liver, spleen, lung, or kidney of the mice treated with PBS or siRNA-loaded NPs. All these results indicate that the NP platform developed in this work could be potentially used as a safe and efficient gene delivery system for GBM treatment.

## Conclusion

In summary, we have developed a robust liposome-based NP platform for systemic siRNA delivery and targeted GBM treatment. This NP platform can target the glioma cells through the specific recognition between their surface-encoded aptide and over-expressed EDB on glioma cells, thereby leading to improved intracellular siRNA delivery and more effective gene silencing in glioma cells. *In vivo* results show that this long-circulating NP platform can target the GBM tumor tissues and obviously inhibit the tumor growth by silencing the CypA expression. Taken together, the NP platform developed herein could potentially serve as an effective delivery tool for non-invasive GBM treatment.

## Author Contributions

PS and XX conceived and designed the experiments. PS, AZ, YN, and YX performed the experiments. PS, YN, LZ, YX, and XX analyzed the data and co-wrote the paper.

## Conflict of Interest Statement

The authors declare that the research was conducted in the absence of any commercial or financial relationships that could be construed as a potential conflict of interest. The handling Editor declared a past co-authorship with the authors PS and XX.
